# RAGE Expression and ROS Generation in Neurons: Differentiation versus Damage

**DOI:** 10.1155/2016/9348651

**Published:** 2016-05-25

**Authors:** S. Piras, A. L. Furfaro, C. Domenicotti, N. Traverso, U. M. Marinari, M. A. Pronzato, M. Nitti

**Affiliations:** ^1^Department of Experimental Medicine, University of Genoa, Via L.B. Alberti 2, 16132 Genoa, Italy; ^2^Giannina Gaslini Institute, Via Gerolamo Gaslini 5, 16147 Genoa, Italy

## Abstract

RAGE is a multiligand receptor able to bind advanced glycation end-products (AGEs), amphoterin, calgranulins, and amyloid-beta peptides, identified in many tissues and cells, including neurons. RAGE stimulation induces the generation of reactive oxygen species (ROS) mainly through the activity of NADPH oxidases. In neuronal cells, RAGE-induced ROS generation is able to favor cell survival and differentiation or to induce death through the imbalance of redox state. The dual nature of RAGE signaling in neurons depends not only on the intensity of RAGE activation but also on the ability of RAGE-bearing cells to adapt to ROS generation. In this review we highlight these aspects of RAGE signaling regulation in neuronal cells.

## 1. Introduction

The receptor for advanced glycation end-products (RAGE) is a multiligand receptor able to bind not only the advanced glycation end-products (AGEs) but also amphoterin, calgranulins, and amyloid-beta peptides (A*β*) [1]. It is normally expressed at low levels in many adult tissues but its activation induces a positive feedback favoring its expression and enhancing cell responses [2]. Through the imbalance of redox state, RAGE activation is involved in the onset and progression of proinflammatory or proapoptotic cell responses [3]. However, more recently the role of RAGE in physiological processes such as cell differentiation has been demonstrated [4]. In this review we focused our attention on the role of RAGE and the associated reactive oxygen species (ROS) production in neuronal differentiation or death.

## 2. RAGE: Structure and Functions

**Figure 1:**
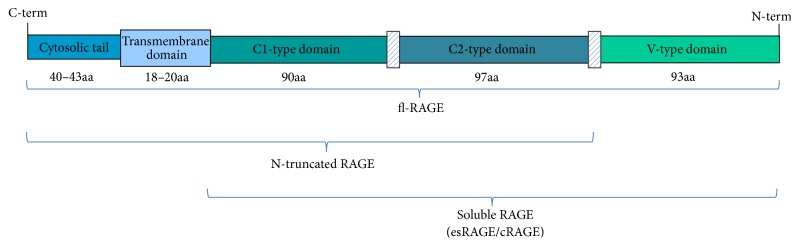
Schematic representation of full-length RAGE and its variants. fl-RAGE is composed of a V-type domain, two C-type domains, a transmembrane domain, and an intracellular tail. The N-truncated form is lacking the N-terminal V-type domain. The soluble form of RAGE is lacking the C-terminal domain but containing all of the immunoglobulin domains. Soluble RAGE may derive from alternative splicing of RAGE mRNA (endogenous secretory esRAGE) or from fl-RAGE proteolytic cleavage from the cell surface (cleaved cRAGE) [13].

RAGE is a member of the immunoglobulin superfamily [5–7]. In humans RAGE gene is localized on chromosome 6, near the major histocompatibility complex III. The gene encodes for a ~55 kDa protein of 404 amino acids [8], the full-length RAGE (fl-RAGE), composed of three structural regions: an extracellular region comprising a V-type domain and two C-types domains, a short transmembrane region, and a cytoplasmic tail [5–7]. The V-type and C1-type domains are the binding sites for the ligands while the 40–43 amino acid cytoplasmic tail is critical for the intracellular signal transduction [2]. In addition, truncated RAGE isoforms have been described. The N-truncated RAGE variant is lacking the N-terminal V-type domain and is localized on the membrane. On the contrary, other variants lacking C-terminal domain but containing all of the immunoglobulin domains are soluble forms of RAGE and secreted extracellularly. Among these soluble RAGE variants, the endogenous secretory RAGE (esRAGE) results from an alternative splicing of RAGE mRNA [9–11], while the cleaved RAGE (cRAGE) derives from fl-RAGE proteolytic cleavage by the metalloproteinase ADAM10 and MMP9 [12, 13] (Figure 1). Soluble forms of RAGE are known to prevent RAGE binding to ligands, acting as a decoy [2].

RAGE expression is dependent on cell type and developmental stage. In general, RAGE is constitutively expressed during embryonic development and downregulated in adult life [14, 15]. Indeed, except for skin and lung where RAGE is highly expressed throughout life, in physiological conditions RAGE is expressed at low levels in a wide range of adult cells such as endothelial cells, cardiomyocytes, neutrophils, monocytes/macrophages, lymphocytes, dendritic cells [15, 16], and, in the adult central nervous system (CNS), glia and neurons [16–19]. However, it has been well shown that RAGE is upregulated in presence of its ligands.

### 2.1. RAGE Ligands

As explained by its name, RAGE has been firstly identified in consequence to its ability to bind to advanced glycation end-products (AGEs) [5–7]. AGEs are a heterogeneous group of compounds, characteristic of aging process and diabetes, which are formed in prooxidant environments, in a time-dependent way, through the nonenzymatic reaction between reducing sugars and free amino residues of proteins [20–22]. The contribution of oxidation is so important that all the process can be referred to as glycoxidative reaction [23]. The glycoxidative damage is a typical hallmark of diabetic sequelae such as nefropathy, neuropathy, or micro- and macrovasculopathies in which the high concentration of blood sugars obviously favors glycative reactions [24]. However, glycative damage plays a key role also in end-stage renal disease associated with uremia and hemodialysis and in different age-related pathologies, especially for the crucial contribution of chronic oxidative damage [25–28]. Later on, several ligands have been found to be able to interact with RAGE, highlighting its multiligand nature [29, 30]. Indeed, HMGB1 (amphoterin), S100/calgranulins, and amyloid-*β* peptides have been identified as ligands of RAGE as well [31].

HMGB1, a highly conserved ubiquitous protein normally expressed in the nucleus is released by necrotic cells and is known to act as a signal of cell damage [32–34]. However, it has been demonstrated that HMGB1 is also secreted by living cells in the central nervous system where it is involved in a number of neuronal functions such as differentiation, cell survival, and neurite outgrowth [14, 17, 35, 36].

S100/calgranulins are a family of calcium-binding polypeptides involved in the regulation of protein phosphorylation, cell cycle, and enzyme activity that accumulate extracellularly in sites of chronic inflammation and act as a proinflammatory stimulus [37, 38].

A*β* peptides derive from amyloid precursor protein (APP) processing and accumulate in Alzheimer's disease, forming the amyloid plaques [39].

Moreover, surface molecules on bacteria, prions, and leukocytes have been demonstrated to be able to interact with RAGE in immune response and chronic inflammation [40–42].

Therefore, the accumulation of all the mentioned ligands leads to the activation of RAGE which not only is involved in the pathogenesis and complications of many aging-related diseases such as diabetes, osteoarthritis, cardiovascular, and Alzheimer's diseases, but also regulates several cellular processes of primary importance such as inflammation, apoptosis, autophagy, and proliferation, playing a crucial role in tissue homeostasis and regeneration [2, 3, 16, 43, 44].

### 2.2. RAGE Signaling

RAGE interaction with its ligands induces different pathways making the RAGE-mediated cellular signaling extremely complex. The activation of a wide array of signaling pathways has been demonstrated: ERK1/2 (p44/p42), p38 and SAPK/JNK MAP kinases, rho-GTPases, phosphoinositol-3-kinase, JAK/STAT, and different PKC isoforms have been shown to play a role in RAGE-mediated cellular responses [2, 45–47]. RAGE-dependent signaling pathway activation directly induces ROS production mainly through NADPH oxidase (NOX) activation, as detailed below. Moreover, it is important to underline that RAGE signaling leads to the activation of the transcription factor NF-*κ*B that in turn induces RAGE expression, making a positive loop that enhances cell response [2]. However, other transcription factors such as SP-1, AP-2, and NF-IL6 have been shown to regulate RAGE expression [2].

#### 2.2.1. RAGE and Oxidative Stress

It is well known that ROS can modulate signal transduction pathways until they are balanced by adequate antioxidant responses but are able to severely damage cells and tissues when redox balance is lost and oxidative stress is induced.* In vitro* studies with cultured capillary endothelial cells and* in vivo* infusion studies have shown that AGE interaction with RAGE leads to oxidative stress, revealed by the appearance of malondialdehyde in the vessel wall and thiobarbituric acid-reactive substances in the tissue [48] and this has been well characterized as one of the crucial mechanism of damage in endothelial cells during diabetes [49].

It has been shown that AGE-RAGE-derived ROS generation is due, at least in part, to the activation of NOX that is able to generate anion superoxide as the main product of its reaction [50, 51]. NOX is a multimeric complex, identified in phagocytes where ROS overproduction leads to bacteria killing [52]. So far, different isoenzymes of NOX have been identified in nonphagocytic cells, active in the generation of ROS for signaling purpose [53, 54] and in neurons NOX1 and NOX2 have been identified [55, 56]. NOX activation leads to NF-*κ*B-mediated iNOS expression favoring the generation of highly toxic peroxynitrite, as shown in vascular smooth muscle cells (VSMC) [34, 57, 58]. However, the controlled ROS production derived from NOX is able to modulate signaling molecules such as p21 contributing to the activation of NF-*κ*B in rat pulmonary artery smooth muscle cells exposed to AGEs [59]. These findings highlight a central role of NOX in the molecular mechanisms involved in RAGE-mediated cell responses.

In addition, the mitochondrial respiratory chain is implicated in ROS generation induced by RAGE activation, as shown with regard to VCAM-1 expression in endothelial cells treated with AGEs [60].

## 3. RAGE and Neuronal Differentiation

RAGE expression in neurons was observed for the first time in adult bovine nervous system mainly in motor and cortical areas [15]. Its identification in normal, nonpathological tissues led the authors to hypothesize a physiological, even though not clear, role played by RAGE. Later on, several studies demonstrated that HMGB1 and S100B, identified as RAGE ligands in the nervous system, are centrally involved in neuronal differentiation and the implication of RAGE in embryonic and adult neuronal differentiation, in peripheral nerve regeneration, and in neurite outgrowth/elongation has been demonstrated [14, 17, 35, 36, 61, 62].

In particular, experimental findings show a functional role of the HMGB1-RAGE-NF-*κ*B axis in the modulation of adult neurogenesis. Indeed, RAGE has been found expressed* in vivo* in the neural stem/progenitor cells (NS/PCs) in the neurogenic Subventricular Zone region of the adult mouse brain where it is coexpressed with Sox2 [63, 64]. However, RAGE expression in mature Tbr1 positive neurons has not been demonstrated, suggesting that RAGE is primarily involved in the early events of adults neurogenesis. In addition, it has been clearly shown that HMGB1 released from reactive astrocytes promotes NS/PCs proliferation through the activation of RAGE and the phosphorylation of JNK [65]. Other studies confirm the crucial importance of RAGE in mediating brain repair and nerve regeneration favoring the crosstalk with inflammatory pathways, as showed by using transgenic mice [61] or in neuronal regeneration induced by S100B [66].

Studies in adult sensory neurons exposed to HMGB1, S100B, or human glycated albumin (HGA) demonstrate that RAGE signaling mediates neurotrophin-dependent neurite outgrowth through the activation of JAK-STAT, ERK, and NF-*κ*B pathways [67]. The RAGE-driven activation of NF-*κ*B in neuronal differentiation and neurite outgrowth has been demonstrated also in Retinoic Acid- (RA-) induced P19 neuronal cell differentiation [68] and in the survival of N18 neuroblastoma and in C6 glioma cells [17]. Furthermore, RAGE, HMGB1, and S100B progressively increase during neuronal differentiation of teratocarcinoma-derived NT2/D1 cells: RAGE is expressed only in cells committed to a neuronal phenotype and directly involved in cellular morphological changes, and S100B seems to be the principal ligand [4]. However, other studies on teratocarcinoma cells and primary neurons show that, although RAGE ectopic overexpression, in absence of RA, is not sufficient to drive neuronal differentiation, cell exposure to RA promotes neurite outgrowth through the activation of RAGE and Rac1/Cdc42 [68]. Moreover, the functional inactivation of RAGE in neuroblastoma cells demonstrates its crucial role in the elongation of neurites rather than in neurite outgrowth [69]. In agreement, our recent study underlines that RA-induced neuroblastoma differentiation promotes RAGE-dependent neurite elongation [70]. In particular, during cell differentiation, A*β*_1-42_ production is increased and, through the binding to RAGE, enhances the expression of the amphoterin-induced gene and open reading frame-1 (AMIGO-1) suggesting its involvement in neurite elongation [70], as also reported by other authors [71–74]. Importantly, we showed that monomeric but no oligomeric A*β*_1-42_ is responsible for this effect, in line with data in the literature sustaining that monomeric A*β*_1-42_ can exert physiological functions while the toxic properties of the peptide are due to its aggregation in oligomers or fibrils [75]. However, the involvement of the oligomeric form of A*β*_1-42_ in neuronal differentiation cannot be ruled out, as shown on hippocampal neuronal progenitors [64] which has been also demonstrated to be dependent on S100B-RAGE interaction [76].

In addition, RAGE activation is able to induce prosurvival signals in neurons. Indeed, HMGB1 and the two S100 family proteins, S100B and S100A1, increase the expression of the antiapoptotic protein Bcl-2, in a RAGE-dependent way, favoring neuroblastoma cell survival [17, 29]. In a similar way, other authors observed that, during RA-induced neurodifferentiation, HMGB1-RAGE interaction is involved in Bcl-2 production [69].

Furthermore, the HMGB1-RAGE interaction induces phosphorylation and nuclear localization of cyclic AMP response element-binding protein (CREB) in ERK1/2 dependent manner, increasing the expression of chromogranins [77] and regulating neuronal differentiation and survival [78].

In addition, several studies clearly show that, in the differentiation of neuroblastoma cells, RA-treatment induces a prooxidative status and modifies gene expression leading to changes in redox environment. In particular, cell exposure to RA increases NOX activity and the mitochondrial membrane potential and, at the same time, induces SOD gene expression, Nrf2 protein synthesis, NF-*κ*B gene expression, and glycolytic pathway upregulation [79–83]. The involvement of ROS in neurite outgrowth and differentiation has been found also in other cell types as primary neurons and pheochromocytoma PC12 cells [84, 85]. Our unpublished results have demonstrated that neuroblastoma cells treated with monomeric A*β*_1-42_ are able to activate NOX favoring neurite elongation (Nitti et al., unpublished).

## 4. RAGE and Neuronal Damage

In addition to its positive effects in neurite outgrowth and neuronal differentiation, RAGE activation can be involved in neuronal damage due to the overproduction of toxic ROS, cytokines and pro-inflammatory molecules [86]. The accumulation of RAGE ligands promotes oxidative stress, progressive neuronal dysfunctions and neurodegeneration. Thus, RAGE-mediated effects are observed in diabetic neuropathy [87] and in the pathogenesis of Alzheimer's [88], Parkinson's [89], Huntington's diseases [90] and amyotrophic lateral sclerosis [91].

Indeed, AGE accumulation and their RAGE-dependent toxic effects on neurons are considered to play a role of primary importance in the pathogenetic mechanism of the diabetic neuropathy and therapeutic approaches against AGE-RAGE have also been proposed [92]. AGEs have been found to accumulate in senile plaques and in neurofibrillary tangles [93] and their ability to activate RAGE contributes to trigger neuronal death during Alzheimer's disease. More recent studies, have shown that genetic deficiency of neuronal RAGE protects against the synaptic injury induced by AGEs in transgenic mice [94]. The direct binding of RAGE to A*β*, mainly to its aggregated forms, is considered important in mediating amyloid toxicity [95] and RAGE activation by HMGB1 has recently been considered to have a crucial role in favoring neurodegeneration contributing to the development of amyotrophic lateral sclerosis [86].

Three main signaling pathways, activated by RAGE in neurodegeneration have been identified: (i) NOX-dependent signaling, leading to ROS production, activating NF-*κ*B and increasing cytokine and chemokine expression; (ii) RAS-dependent signaling, activating MAP kinases (JUN, ERK1/2 or p38) and modulating NF-*κ*B; (iii) JAK/STAT signaling, leading to the induction of interleukin expression. In all cases, RAGE activation favors the generation of ROS from mitochondria, induces protein aggregation and increases the release of pro-inflammatory molecules [86]. It is important to note that RAGE is also expressed on microglial cells, where it plays a crucial role enhancing cytokine production, oxidative stress and neuroinflammation [96–98].

In addition, it has been demonstrated that RAGE mediates A*β* transport via endocytosis and transcytosis across the blood-brain barrier (BBB), promoting A*β* pathologic accumulation in brain parenchyma [99, 100]. Interestingly, RAGE can act as a carrier for A*β* also on neuronal cell surface: RAGE-dependent p38 MAPK activation promotes the internalization of the whole A*β*-RAGE complex into the cytosolic compartment, leading to mitochondrial dysfunctions, oxidative stress, and neuronal damage [101].

Moreover, RAGE activation can induce neuronal loss triggering the apoptotic process and, in some cases, inducing ER-stress, or favoring autophagy. Indeed, it has been demonstrated that HMGB1-RAGE interaction induces neuronal apoptosis in mixed neuron-glia cultures via p38 MAPK and ERK signaling activation [102]. Similarly, RAGE-dependent apoptosis has been described also in neuroblastoma cells exposed to S100B or AGEs [17, 103]. Moreover, S100B production and subsequent RAGE expression can lead to neuronal apoptosis mediated by ER-stress in infantile neuronal ceroid lipofuscinosis (INCL) and palmitoyl-protein thioesterase-1- (PPT1-) KO mice [104–106]. Furthermore, A*β*-RAGE interaction can increase intracellular Ca^2+^, that, activating CaMKK*β*-AMPK, leads to autophagosome formation in neuroblastoma cells, hypothesizing the involvement of autophagy in A*β*-dependent neurodegeneration [107].

## 5. Conclusions

**Figure 2:**
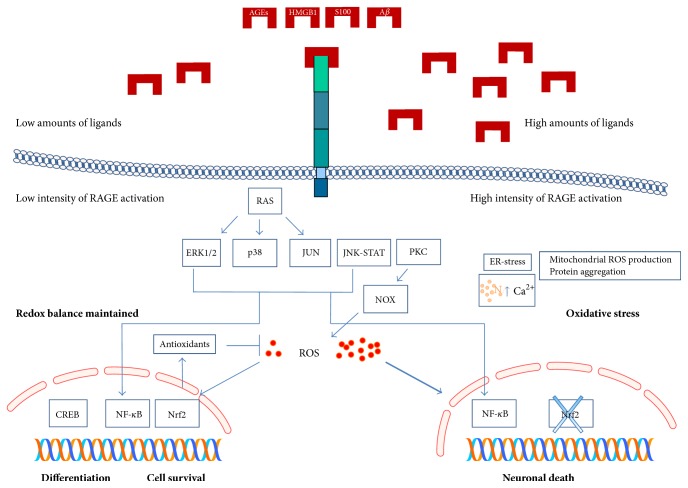
Schematic representation of RAGE signaling in neurons. Differences between prodifferentiating pathways and death signals are highlighted.

In this review we have shown the double nature of RAGE in neuronal cells: on the one hand, it is able to increase cell survival and favor neuronal differentiation; on the other hand, its activation induces neuronal death. These contradictory effects seem not to be related to the ligand of RAGE, as we provided evidence that the different ligands are able to induce both kinds of cell response but probably depend on the intensity and duration of RAGE activation and, crucially, on specific features of RAGE-bearing cells. Indeed, it has clearly shown that low-level of RAGE activation, induced by low concentration of ligands, has prosurviving differentiating effects, while, in the presence of high amounts of ligands, RAGE induces neuronal death [17]. Interestingly, it has been proved that neurons can be preconditioned by low-level RAGE stimulation increasing their resistance to the toxic effects of high concentrations of RAGE ligands. Indeed, ROS derived by RAGE activation seem to play a crucial role due to their ability to activate prosurviving NF-*κ*B-dependent pathways when they are generated in low amounts [108, 109]. However, in order to balance ROS production and counteract oxidative stress, the production of molecules with antioxidant and detoxifying activities, such as glutathione or heme oxygenase-1, becomes a key point in neuronal response to RAGE activation. Therefore, when RAGE-expressing cells are able to induce a balanced antioxidant response (e.g., in undifferentiated cells) ROS generation can be kept at low levels acting as molecular mediators of cell growth and differentiation. On the contrary, when cells are unable to properly adapt to ROS generation (e.g., in fully differentiated cells or in aging neurons), RAGE activation induces oxidative stress leading to neuronal death (Figure 2). Our previous studies demonstrated that neuroblastoma cells, basically resistant to AGE exposure, become sensitive to AGEs only after cell differentiation [110] and also fully differentiated NT2 neurons, unable to react to RAGE-dependent ROS generation, are sensitive to glycated serum [45]. It is conceivable that the transcription factor Nrf2, master regulator of antioxidant and adaptive response, plays a role in the neuron response (differentiation or death) after RAGE activation. Indeed, it has recently shown that Nrf2-dependent responses are necessary to complete the differentiation program, whilst in terminally differentiated neurons the impairment of Nrf2 signaling is involved in the enhancement of neuronal sensitivity to oxidative stress [111]. This becomes particularly important in aging which is known to further impair the function of Nrf2 [112] and seems to be important in neuronal response to RAGE activation. It has been recently clearly demonstrated that pharmacological approaches able to inhibit RAGE activation and to stimulate Nrf2 activity, reducing oxidative stress, improve learning and memory in AD mice [113]. These findings underline that the ability to adapt to ROS generation is a crucial point in defining neuronal response to RAGE activation.
